# Data Linkage: A powerful research tool with potential problems

**DOI:** 10.1186/1472-6963-10-346

**Published:** 2010-12-22

**Authors:** Megan A Bohensky, Damien Jolley, Vijaya Sundararajan, Sue Evans, David V Pilcher, Ian Scott, Caroline A Brand

**Affiliations:** 1 Centre of Research Excellence in Patient Safety, Dept of Epidemiology & Preventive Medicine, School Public Health & Preventive Medicine, Monash University, Melbourne, Victoria, Australia, 3181 http://www.crepatientsafety.org.au; 2 Department of Health Victoria, 50 Lonsdale Street, Melbourne Victoria, Australia 3000; 3 Australian & New Zealand Intensive Care Society, Centre for Outcomes and Resource Evaluation, 10 Ievers Terrace, Carlton Victoria, Australia 3053; 4 Department of Internal Medicine, Princess Alexandra Hospital, Brisbane, Queensland, Australia 4102

## Abstract

**Background:**

Policy makers, clinicians and researchers are demonstrating increasing interest in using data linked from multiple sources to support measurement of clinical performance and patient health outcomes. However, the utility of data linkage may be compromised by sub-optimal or incomplete linkage, leading to systematic bias. In this study, we synthesize the evidence identifying participant or population characteristics that can influence the validity and completeness of data linkage and may be associated with systematic bias in reported outcomes.

**Methods:**

A narrative review, using structured search methods was undertaken. Key words "data linkage" and Mesh term "medical record linkage" were applied to Medline, EMBASE and CINAHL databases between 1991 and 2007. Abstract inclusion criteria were; the article attempted an empirical evaluation of methodological issues relating to data linkage and reported on patient characteristics, the study design included analysis of matched versus unmatched records, and the report was in English. Included articles were grouped thematically according to patient characteristics that were compared between matched and unmatched records.

**Results:**

The search identified 1810 articles of which 33 (1.8%) met inclusion criteria. There was marked heterogeneity in study methods and factors investigated. Characteristics that were unevenly distributed among matched and unmatched records were; age (72% of studies), sex (50% of studies), race (64% of studies), geographical/hospital site (93% of studies), socio-economic status (82% of studies) and health status (72% of studies).

**Conclusion:**

A number of relevant patient or population factors may be associated with incomplete data linkage resulting in systematic bias in reported clinical outcomes. Readers should consider these factors in interpreting the reported results of data linkage studies.

## Background

Reports in the United States [1], Canada [2], United Kingdom [3] and Australia [4] have recommended increasing the use of existing data, such as administrative and clinical registry data, to provide comparative clinical performance data to health services, hospitals, clinical units and clinicians via internal channels and to consumers via publicly accessible media. Although a limited number of patient outcomes, such as in-hospital mortality, complication and re-admission rates are currently available from some administrative data sources, obtaining data from several different databases pertaining to the one individual or participant using data linkage is often necessary to ensure adequate risk-adjustment and examine a more comprehensive range of outcomes for comparison between organisations.

Data or record linkage has been defined as "a process of pairing records from two files and trying to select the pairs that belong to the same entity [5]." Linkage may be conducted between two distinct data sources or within a single data-set to identify multiple entries (e.g. re-admissions) for one person or record unit. The various uses of linked data in clinical research and the types of data linkage that can be deployed are briefly described in additional file 1. In the United Kingdom, 47% of multicentre clinical databases surveyed in 2003 by Black *et al *reported that they undertook routine data linkage to other databases [6]. A review by Evans *et al *reported that 68% of Australian clinical registries routinely undertook some form of data linkage to obtain outcome information, such as death or disease status, and to assess data quality [7]. The use of data linkage in research studies has increased almost 6 fold within the last two decades. A search by one of the authors (MAB) of the term "data linkage" and of the heading "medical record linkage" in study abstracts and titles on Medline identified only 161 studies between 1992 and 1997, compared to 951 studies between 2002 and 2007. This proliferation of data linkage is reflected in the establishment of data linkage research centres and initiatives in Australia [8,9], North America [10,11] and the United Kingdom [12,13].

While data linkage is an important tool in observational research, it may be associated with various types of error. When linking two data-sets, there is a proportion of cases that will match and a proportion that will remain unmatched. Error arises if data sources do not consistently capture the same cases, records that correspond to the same person do not link due to missing or inaccurate data (false negatives), or unrelated records are mistakenly linked (false positives).

It is often difficult to assess the quality of a linkage when the patient outcome which is being linked to other variables is unknown or there is not an expected one-to-one relationship between one variable and another. For example, when linking records to a death registry to determine a patient's survival status, it is difficult to know which matches have been missed if it is unknown whether the patient is alive or dead. A systematic review of probabilistic linkage accuracy identified only six articles that had complete data on summary measures of linkage quality and found the sensitivity of linkage (ie the proportion of truly matched records detected) to range from 74% to 98% and specificity (ie the proportion of truly unmatched records detected) to range from 99 to 100% [14]. In the studies with lower sensitivity, findings may be biased leaving the results and their interpretation open to question.

There is a need for clinicians and policymakers to understand the limitations of linkage for outcome measurement. Methods for evaluating the quality of existing data sources and operating data linkage services have been previously reported [15-18]. While these publications offer a framework for evaluating existing data sources and managing data linkage services, there is a paucity of literature that discusses the quality and limitations of research using linked data.

The purpose of this study was to synthesize the evidence through a structured narrative review of patient or population characteristics that may be associated with changes in sensitivity and specificity of data linkage, thereby introducing systematic bias into reported outcomes.

## Methods

A structured, narrative review of the literature was considered appropriate for this form of observational research. Medline, EMBASE and CINAHL databases were search using the search strategies reported below:

• MEDLINE

"data linkage" as keywords in title, abstract, name of substance word, subject heading word

"medical record linkage"[Mesh]

Limited to years 1991-2007, the English Language and studies involving humans.

• CINAHL

Data linkage OR Medical record linkage

Limited to years 1991-2007, the English Language and studies involving humans

• EMBASE

Data AND Linkage

Medical AND Record AND Linkage

Limited to years 1991-2007, the English Language and studies involving humans, excluding letters, notes, commentaries and editorials.

*A priori *criteria for abstract inclusion were that the article empirically evaluated methodological issues relating to data linkage and reported on patient characteristics in matched versus unmatched records. Articles were excluded if they did not involve data linkage, such as a discussion of another form of health information technology; if they did not involve data, such as a commentary, letter or discussed a data linkage methodology without an empirical evaluation or if they presented a linkage project without comparing characteristics in matched and unmatched records. We excluded studies with an area of focus in genetics from our EMBASE search, as our search term was detecting genome linkage projects. We reviewed a subset of these studies (n = 105, 10%) to determine if any of these articles met our study inclusion criteria and confirmed they did not.

Study titles, abstracts and full articles were screened and the review and evaluation of the studies were performed by one researcher (MAB). Participant characteristics influencing the completeness of data linkage were grouped thematically after reviewing studies that met the inclusion criteria. As no quality grading tool exists for studies utilising linked data, we assessed the quality of the studies by examining the participant characteristics that were assessed and the methods used for evaluating potential sources of bias.

## Results

**Figure 1:**
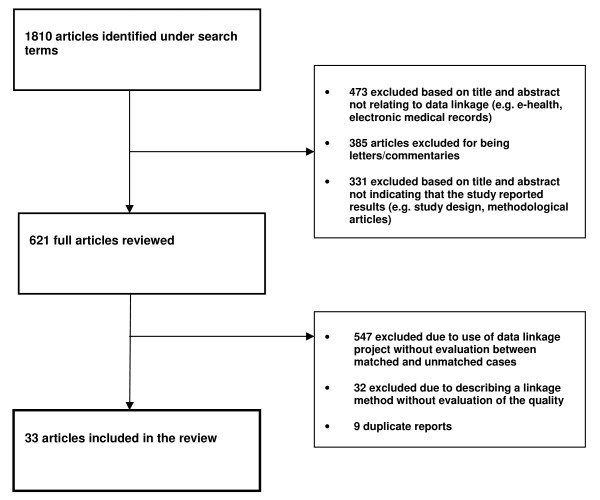
**Flow diagram of included and excluded studies**.

Using the search strategy described above, the search of Medline identified 1451 articles, CINAHL identified 317 articles and EMBASE identified 42 articles not identified in the MEDLINE searches. Of the 1810 studies identified in the search, 1416 abstracts (78.2% of all studies) were screened. Of these, 33 (1.8%) articles met the inclusion criteria (see Figure 1 for search process). A review of the references of the included studies did not identify any additional articles meeting our inclusion criteria. All articles were grouped thematically according to patient or population characteristics that were compared in matched and unmatched records (listed in additional file 2). Below, we describe characteristics of the identified studies and present a qualitative synthesis of the differences in matching rates according to each of the identified patient or population characteristics: age, sex, ethnic/racial group, geographical/hospital site, socio-economic status and health status. Selected case study descriptions are provided in additional file 3.

### Study characteristics

While a number of studies used data linkage methods (n = 612), we identified only 33 studies that examined characteristics of matched versus unmatched records. Identified studies were from a range of countries (12 from the United States, 8 from Australia, 5 from the United Kingdom, 4 from Canada and 1 each from the Netherlands, New Zealand, Switzerland and Taiwan) demonstrating the increasing popularity of these methods. Among the included studies, there was broad heterogeneity of reported characteristics. Some studies only reported one patient characteristic while others reported up to six. The methods for assessing the completeness of linkage were also inconsistent. Some studies compared proportions of different patient characteristics among matched and unmatched records, while others examined the odds of a successful match using regression analysis. The influence of missed links on the measure of association between exposure and outcome is difficult to quantify where data are unlinked (i.e. if the data with the exposure measure are not linked to the corresponding data with the outcome of interest). However, some studies did attempt to account for this through a sensitivity analysis of possible outcomes. Common reasons for the linkage errors have been categorised in additional file 4 along with a framework for potential reporting items that can assist in developing greater standardisation in this area.

### Age

Eighteen studies looked at the relationship between participants' age and linkage rate. Five of these studies found that no significant relationship between age and linkage errors [19-23]. There was a trend for older participants to be less likely to consent to record linkage [24-26]. Where consent was not sought, the association between age group and correctly matched records was less clear with both younger and older participants having lower linkage rates [27-35]. Adams *et al *found that mothers aged under 18 years were more likely than other age groups to have missing social security numbers (21.4% vs 3.2% for 25-29 year olds; p < 0.00), which was a key linkage variable used to match their records to their child's birth records [27]. As younger maternal age has been associated with greater risk of adverse pregnancy outcomes, the higher potential for missed linkage when relying on social security number could underestimate this as a risk factor.

### Gender

Ten of the identified articles compared gender in linked and unlinked records. In five of these articles, the relationship was not found to be significant [19,21,22,25,31]. In three studies, males had lower linkage rates [24,28,29] and in two studies women had lower linkage rates [34,35]. Males were found to be less likely to consent to record linkage in one study [24]. As women's names are more likely to change over their lifespan, linkage relying on surnames may explain the discrepancy in at least one of the studies [34]. In the other study, the linkage utilised participants' social security numbers, which more women than men may not have if they have never been employed [35].

### Ethnic/racial groups

Differences in linkage rates among ethnic or racial groups were examined in fourteen studies with five finding no variations in rates of linkage error according to ethnic group [21,23,25,34,36]. In seven studies, it was found that people in minority groups had lower linkage rates [26,27,35,37-40]. Several studies offered reasons for this finding including subjects in minority groups being treated at facilities with less complete data [38], a lower rate of migrants reporting accidents to the police [40], higher rates of non-consent to data linkage [26] and lower likelihood of having social security numbers recorded [35]. Conversely, in one Australian study, Australian-born mothers had lower linkage rates due to more frequent treatment in private hospitals which lacked data on mothers' full names [20]. The remaining study found that estimations of death varied for ethnic groups depending on the linkage method [30].

### Geographical/hospital sites

As data collection practices and training are likely to vary between sites, 13 of 14 identified studies found a relationship between the quality of linkage at different geographic or hospital sites [25-27,29,33,34,38,40-45] with only one study finding no relationship [21]. Darlymple *et al *examined the use of a record linkage system to track and identify treatment patterns of patients with psychiatric conditions in Ontario, Canada. It was found that the prevalence of linkage error in the community health sector was five times that of psychiatric hospitals, possibly due to a higher turnover of data entry staff with less training [42]. Within community agencies, misidentification by unique identifier code was approximately 22% compared to 0.5% for psychiatric hospitals, making it difficult to accurately track utilisation patterns of patients treated outside of psychiatric hospitals.

### Socio-economic status

Eleven articles examined socio-economic status, and other proxy measures of social deprivation (assessed by income level, car availability and insurance status) and linkage rates. Two studies did not find a relationship [23,41], while nine studies found people in lower socio-economic groups and with lower levels of educational attainment were less likely to have matched records [21,25-27,32,34,37,46,47]. The differences were mainly attributed to lower consent rates for linkage [21,25,26,47,48] and less complete data and reporting for people in lower socio-economic categories [36,49].

### Health status

Eighteen studies were identified that examined health status in matched and unmatched records. Five of the eighteen studies that examined the relationship between health status or condition severity found that differences in health status were evenly distributed between matched and unmatched records [19,25,39,50,51]. Tromp *et al *found that twin births with multiple readmissions had errors in the linkage of readmission records due to inaccuracies in twin rank data [51]. Generally, people with poorer health tended to have higher rates of consent for record linkage [21,24,47] and five studies found that pre-term and low birth weight children tended to be under-reported or have less accurate data [20,36,45,49,52,53]. Three studies found patients with greater illness or injury severity had better quality data, as a result of being treated in hospital as opposed to lower acuity health services [31,40,42]. Kariminia *et al *found that the sensitivity of reporting drug-related deaths and suicides was lower compared to other causes of death [54], while Magliano *et al *found that reporting of cancer-related deaths was higher than deaths due to cardiovascular disease [55].

## Discussion

The results of this review have identified a number of participant characteristics (age, gender, race, setting, socioeconomic and health status) that are associated with incomplete data linkage and potential for systematic bias in reported outcomes. As demonstrated in additional file 2 there is heterogeneity of reported patient characteristics and methods for assessing and reporting these differences, which highlights the lack of standardisation in this area. To our knowledge, this is the first study to synthesize the literature on assessing quality of data linkage and suggests how it may influence the validity of research results. For many variables examined in the reported studies, the evidence for an association between the variable and rates of data linkage was inconsistent. This may reflect differences in sampled populations and other contextual factors.

The reasons for different rates of completeness in data linkage are varied but can be broadly grouped into the methods of linkage, governance issues, such as requiring consent for linkage, and the accuracy and completeness of the data within each data source. These may be general issues related to the linkage variables, such as differences in linkage rates by gender resulting from female's changing their names, or contextual issues related to a specific study or setting, for example the use of social security numbers as a linkage variable in the United States. In countries that lack a national health identifier, data linkage commonly relies on names or medical record numbers, and these may be collected with varying degrees of accuracy and completeness across hospital sites, as was identified in the study by Ford *et al *[20].

This study has limitations that should be noted. There is currently no grading system available to assess studies utilizing linked data. Existing critical appraisal tools, such as the STROBE guidelines[56], do not address issues of bias associated with data linkage. Consequently, we were unable to apply a validated, standardized tool to assess the quality of these studies. However, this review was intended to be an exploratory discussion of potential sources of bias to highlight these issues for researchers and encourage more systematic assessment and reporting of linkage methods in the future. In addition, a proportion of articles (52%) were excluded after reviewing the abstracts, as they did not appear to discuss data linkage (e.g. articles about electronic medical records, e-health) or they commented on methodological issues related to data linkage without an empirical study. This may mean that some articles meeting the inclusion criteria were missed if the abstract lacked detail. However, full articles were sought for all articles where the study title and available information appeared to be related to data linkage but lacked a detailed abstract.

When linking existing data sources to measure outcomes of care, such as mortality rates, differences such as those described above could underestimate mortality for participants in any of the groups identified, thereby skewing perceptions of quality of care. Linkage issues may be compounded by broader data quality issues, such as a lack of standardised data definitions and inconsistent coding practices [57], which will also compromise quality of measurement. While this paper has focused on data linkage, it is important to consider the influence of data linkage in the broader context of other data quality issues when using existing data to monitor quality of care.

To accurately assess for bias due to errors in linkage, characteristics of unmatched records and a measure of the quality of linkage, such as the rates of false positives and false negatives, need to be routinely measured and reported. Depending on the research question and outcomes under study, potential bias in study results needs to be assessed on a case-by-case basis. Biased estimation of outcomes in either direction will have negative consequences for measuring, and therefore improving, the quality of health care. Furthermore, unreliable and invalid data will continue to undermine the confidence of clinicians and other stakeholders in measurement systems relying on existing data sources.

As a way forward, in additional file 4 we have presented the different reasons for unlinked records and suggested some factors that should be considered in the evaluation of studies using linked data in assessing the quality of data linkage. Studies should clearly describe the data-sets being used, the linkage variables and process and an assessment of the quality of the linked data-sets to identify potential sources of bias. Even where unique identifiers exist, cases may not link due to missing or inaccurate data or inconsistent data definitions, inclusion criteria or coding practices. It can be difficult to determine the impact of these issues when the quantity of missing data and unlinked records are not reported. An appropriately validated quality assessment tool specific to data linkage studies would help to systematically identify and review the specific sources of bias discussed in this paper. Such a tool would assist clinicians and policy-makers in interpreting the findings of clinical research studies based on data linkage and encourage more consistent reporting by researchers.

## Conclusions

Studies reliant on linked data may yield biased findings due to errors in data linkage which relate to specific characteristics of patients and clinical settings. Such errors may not be readily apparent due to inconsistencies in the way researchers evaluate and report them. If existing data sources and linkage processes are to be used for assessing quality of care, it is important that clinicians and policymakers recognise their limitations in order to avoid misinterpreting their findings. Reporting and appraising results based on linked data in a transparent and consistent manner will help to highlight limitations of current healthcare data sources and assist in improving data collection, coding practices and linkage processes.

## Competing interests

The authors declare that they have no competing interests.

## Authors' contributions

MAB conducted the searches, analysed the data and wrote the article, CAB supervised data analysis and reviewed the article, DJ, VS, SE, DVP and IS, supervised and reviewed the article. All authors read and approved the final manuscript.

## Pre-publication history

The pre-publication history for this paper can be accessed here:

http://www.biomedcentral.com/1472-6963/10/346/prepub

## Supplementary Material

Additional file 1 **Linkage methods**. A description of the principles of data linkage and the methods commonly undertaken. Click here for file

Additional file 2 **Selected Case Studies**. Several studies are highlighted to demonstrate how linkage error can influence findings when applied to health services research. Click here for file

Additional file 3 **Summary of Study characteristics associated with unmatched data linkage cases**. A table of each study included in the review and how selected population characteristics were influenced by unmatched records. Click here for file

Additional file 4 **A framework for evaluating data linkage studies**. Reasons for unlinked records and suggested reporting items for studies utilising data linkage to identify potential quality issues. Click here for file
