# Supplementary material for: Data Linkage: A powerful research tool with potential problems
Source: BMC Health Serv Res. 2010 Dec 22;10:346. doi: 10.1186/1472-6963-10-346 (PMC3271236; doi:10.1186/1472-6963-10-346)
Supplement: Additional file 1 — Linkage methods. A description of the principles of data linkage and the methods commonly undertaken. [file 1472-6963-10-346-S1.DOC]

Additional File 1. Linkage methods

## In health care evaluation, data linkage can enable researchers to measure care across sectors, assess the integration of care, consider long-term patient outcomes, monitor service provision, identify adverse outcomes and compare data obtained from different sectors or agencies. Examples of health services evaluation using linked data include studies into the long-term survival rates of intensive care patients , the association between performance measures and outcomes following hospitalisation heart failure , and studies of adverse drug reactions in the elderly .

When linking data-sets, there are two commonly used methods: deterministic and probabilistic linkage. Deterministic linkage is applied when there are one or several identifiers, such as a health care service number (e.g. NHS number or US Medicare number) or a national identity number that will match completely with the other data-set(s). The identifiers may be used alone or in combination.

Probabilistic linkage is typically used when a unique identifier is not available and involves the matching of partially identifying variables, which may not be unique. Agreement and disagreement weights for each variable are calculated according to standardized formulae.^14^ Thresholds of certainty around whether records are truly matched can be pre-determined by the analysts to decide which links will be accepted as true matches and which will be rejected. The error rates can be pre-specified by analysts, but involves a trade-off between precision and sensitivity of data linkage.

A combination of deterministic and probabilistic methods can also be used.
