# Supplementary material for: Data Linkage: A powerful research tool with potential problems
Source: BMC Health Serv Res. 2010 Dec 22;10:346. doi: 10.1186/1472-6963-10-346 (PMC3271236; doi:10.1186/1472-6963-10-346)
Supplement: Additional file 2 — Selected Case Studies. Several studies are highlighted to demonstrate how linkage error can influence findings when applied to health services research. [file 1472-6963-10-346-S2.DOC]

Additional File 2. Selected Case Studies

CASE 1

Study Findings

Darlymple *et al* found errors in linkage variables were 5 times more prevalent at community mental health centres than at psychiatric hospitals, with the latter tending to have dedicated data entry staff [[42](#_ENREF_42)].

Impact for Health Services Research

The accuracy of long-term utilisation patterns and outcomes of patients receiving community mental health services will be biased towards the null due to the poorer quality data that is being collected and linked. As many patients receive mental health care in both the community and acute sectors, this will make it difficult to track patients’ progress over the full duration of their illness.

CASE 2

Study Findings

Liu *et al* found babies with jaundice or low birth weights were significantly more prevalent in unmatched cases compared to those without jaundice or with normal birth weight [[53](#_ENREF_53)].

Impact for Health Services Research

As this data-set was being linked to study the relationship between length of stay and re-admission patterns of neonates, the differences in rates of linked and unlinked cases between the two groups of babies may bias any difference in readmission rates between the two groups towards the null.

CASE 3

Study Findings

Zingmond *et al* linked ten years of hospital discharge data in the state of California to death records using the United States social security number. Females, the elderly, Hispanics and non-Hispanic Blacks were less likely to have social security numbers and, therefore, had higher proportions of unlinked death records [35].

Impact for Health Services Research

As some traditionally vulnerable populations are more likely not to have social security numbers, this method of linkage can underestimate mortality outcomes.
