# Supplementary material for: Data Linkage: A powerful research tool with potential problems
Source: BMC Health Serv Res. 2010 Dec 22;10:346. doi: 10.1186/1472-6963-10-346 (PMC3271236; doi:10.1186/1472-6963-10-346)
Supplement: Additional file 3 — Summary of Study characteristics associated with unmatched data linkage cases. A table of each study included in the review and how selected population characteristics were influenced by unmatched records. [file 1472-6963-10-346-S3.doc]

Additional File 3. Summary of study characteristics associated with unmatched data linkage cases*

| Study, Year | Result |
| --- | --- |
| Age | |
| 1. Adams MM 1997[27] | Key linkage variables had a completeness rate of 78.6% for people under 18 years and 96.8% for 25-29 year olds. |
| 2. Baker R 2000 [19] | Asthmatic patients who consented had a mean age of 47.5 v 45.7 years of non-consenters. Patients with angina who consented had a mean age of 68.8 v. 69.2 years. Differences were not significant. |
| 3. Blakely T 2000 [37] | Linkage rates were 52.1% for males & 58.8% for females among 15-24 year olds and 81.3% males and 79.7% females among 65-74 year olds. |
| 4. Bopp M 2003 [29] | 24% of records were unlinked among 25-34 year olds. Rates were not reported for other age groups but were noted to be lower. |
| 5. Buescher PA 1999 [30] | 5 per 1000 deaths were identified among infants aged 28 days or less and 7.2 per 1000 death depending on linkage methods. Mortality rates were more constant for other age groups. |
| 6. Cryer 2001 [31] | 89.3% records were linked among people under age 16 and. 99.3% among people aged 25-34 years. |
| 7. Dunn KM, 2004 [24] | 38-69% consented to linkage among females aged 18-29 across studies compared to 67-84% among females aged 60-69 years. |
| 8. Ford JB 2006 [20] | Mean age was 29.6 years in matched pairs and 28.9 in unmatched pairs. These differences were not significant. |
| 9. Harris T 2005 [21] | The Odds ratio for consent given was 0.9 (95% CI: 0.6 to 1.5) for those aged 70-79 and 0.8 (95% CI: 0.5 to 1.3) for those aged 80 plus compared to people aged 65-69. |
| 10. Holian J 2000 [36] | 0.5% records were missing mother’s age among linked records compared to 25% among unlinked records. |
| 11. Huang N 2007 [25] | 9.3% of people aged 20-24 did not provide consent compared to18.3% of people aged 75 plus. |
| 12. Jebamani LS 2005 [33] | People aged 35-54 had a linkage rate of 57% compared to 50% among 90 plus year olds. |
| 13. Maizlish NA 2005 [34] | Matching sensitivity was 96.7% for <25 year olds and 91.1% for 45-64 year olds (p<0.05) |
| 14. Nitsch D 2006 [41] | Births occurring in the earliest years of the registry were more likely to be missed. |
| 15. Sundararajan V 2004 [22] | Proportions were not reported but noted to be non-significant differences in matched compared to unmatched age groups. |
| 16. Tate AR 2006 [26] | Non-consent was 7.0% for mothers aged 41yrs and 4.1% for mothers aged 14-20 yrs. |
| 17. Waller DK 1996 [23] | Proportions were not reported but age distributions were noted to be identical in matched compared to unmatched records. |
| 18. Zingmond DS 2004 [35] | Unmatched records ranged from 10-29% for women under 1 year of age to. 3-5% for 35-64 year-olds across race strata. |
| Gender |  |
| 1. Baker R 2000 [19] | 9.9% of males with asthma did not consent to linkage compared to 10.8% of females. 8.0% of males with angina did not consent to linkage compared to 10.7% of females. These differences were not found to be significant. |
| 2. Blakely T 2000 [37] | Males had a correct linkage rate of 75.7% compared to females 77.9%. |
| 3. Bopp M 2003 [29] | 13% of women’s records remained unlinked. The proportion was noted to be lower for men but no exact measure was provided |
| 4. Cryer PC 2001 [31] | 36% of the linked cases were female compared to 37% females in all hospital admissions. This was not found to be significant. |
| 5. Dunn KM 2004 [24] | Among people aged 40-49, 49% of men consented to linking their survey data to medical records compared to 67% of females. This trend was consistent until aged 70 when both genders consented at similar rates. |
| 6. Harris T 2005 [21] | Females had an odds ratio for giving consent of 0.8 (95% CI: 0.5 to 1.1). |
| 7. Huang N 2007 [25] | Females had and adjusted odds ratio for giving consent of 1.02 (95% CI: 0.92 – 1.14). |
| 8. Maizlish NA 2005 [34] | The sensitivity of matching was 94.2% among males v. 91.6% among females. (P< 0.05) |
| 9. Sundararajan V 2004 [22] | Proportions not reported but noted to be non-significant differences in matched compared to unmatched gender distribution. |
| 10. Zingmond DS 2004 [35] | Among people aged 65-84 years, unmatched record rates were 4-8% for females across race strata v. 2-4% for males. |
| Race/Ethnicity |  |
| 1. Adams MM 1997 [27] | 12.6% of women from “other” races (i.e. not Black or white) were missing key linkage variables v. 5.8% of white women. |
| 2. Blakely T 2000 [37] | There was a 63.4% linkage rate for Maori ethnicity, 57.7% for Pacific ethnicity, 78.5% for non-Maori, non-Pacific, and 81.9% for people with no specified ethnic group. |
| 3. Buescher PA 1999 [30] | Infant (<1 yr) mortality rate ranged from 10.7 per 1000 births v. 14.0 per 1000 births for minority ethnic groups between two linkage methods. Linkage rates were more constant across other ethnicities. |
| 4. Ford JB 2006 [20] | 72.6% of linked records were for women born in Australia v. 77.9% of unlinked records, demonstrating an uneven distribution. |
| 5. Gyllstrom ME 2002 [38] | Hispanic women had a linkage rate of 85.6% v 92.5% for White women. |
| 6. Harris T 2005 [21] | Non-whites had an odds ratio for giving consent of 0.9 (95% CI: 0.4 to 2.3) compared to white people. |
| 7. Holian J 2000 [36] | 33.6% of the linked cohort was white compared to 30% of unregistered patients. 65.9% of the linked cohort was black compared to 70% of unregistered patients. These differences were not found to be significant. |
| 8. Huang N 2007 [25] | Indigenous (OR=0.23, 95% CI:0.12-0.42), Hakka (OR=0.90, 95%CI: 0.75 – 1.08) and people of “Other” races (OR=1.05, 95% CI: 0.89 – 1.25) had slightly different adjusted odds for providing consent compared to Fujianese people. |
| 9. Maizlish NA 2005 [34] | Sensitivity of linkage ranged from 87% in White people to 94% in African American people. This difference was not found to be significant. |
| 10 Ringland C 2006 [39] | Sensitivity of the matching process was reported to be higher for those who spoke English at home compared to those who did not. |
| 11. Rosman DL 1994 [40] | Asian born persons had linkage rates of 55% v 70% for European-born persons (p<.001). Aboriginals had linkage rates of 48% v. 64% for non-Aboriginals (p<.001). |
| 12. Tate AR 2006 [26] | Non-consent to linkage was highest among mothers of Bangladeshi origin at 21.5% v. 4.6% for mothers of British/Irish white origin. It was consistently higher across other minority ethnic groups as well (11.3 - 17.8%). |
| 13. Waller DK 1996 [23] | Proportions were not reported but race/ethnicity distributions were noted to be identical in matched compared to unmatched records. |
| 14. Zingmond DS 2004 [35] | Across age/sex strata unlinked records ranged from 2-10% among Asians v. 3-21% among Hispanics. |
| Geographical/Hospital Site |  |
| 1. Adams MM 1997 [27] | 16.7% of mothers born overseas were missing key linkage variables v. 5.9% of mother’s born in the state of Georgia (where the study took place). |
| 2. Bopp M 2003 [29] | 27% of people from larger cities had unlinked records. This was noted to be a higher proportion than for people inhabiting other areas. |
| 3. Cote TR 1995 [43] | Completeness of case ascertainment of people with AIDS ranged from 77-93% by geographical region in the AIDS registry and 68-100% in the cancer registry. |
| 4. Darlymple AJ 1994 [42] | Within community agencies, misidentification by unique identifier code was approximately 22% compared to 0.5% for psychiatric hospitals |
| 5. Gyllstrom ME 2002 [38] | Unmatched records ranged from 72-97% in four border counties v. 0-24% in most other counties. |
| 6. Harris T 2005 [21] | People attending the suburban primary care clinic had odds of 0.8 (95% CI:0.5 to 1.1) for providing consent compared to those attending the urban clinic, although the difference was not found to be significant. |
| 7. Hoving JL 2005 [44] | Under-ascertainment of death linkage was noted to be 13% in the state of Victoria and 11% in the state of Western Australia. |
| 8. Huang N 2007 [25] | Residents of suburban areas were less likely to consent to record linkage (84.9%) than rural/urban residents (88.4-89.6%). |
| 9. Jebamani LS 2005 [33] | Rates of unlinked records varied from 37- 50% in different health authority regions. |
| 10. Maizlish NA 2005 [34] | The sensitivity of matching varied by health centre from 79.2-94.9% (p<0.05). |
| 11. Nitsch D 2006 [41] | 24% of the missed links were for births occurring outside of Scotland. |
| 12. Rosman DL 1994 [40] | Patients admitted to private hospitals had linkage rates of 30.7% v. 71.6% for patients admitted to metropolitan teaching hospitals. |
| 13. Sunderam S 2006 [45] | Patients residing in the state of Massachusetts had a linkage rate of 92.2% v. 85.5% for those whose state of residence was unknown (p<0.01). |
| 14. Tate AR 2006 [26] | 5.3% of people from England did not give consent v. 10.9% from Northern Ireland. |
| SES |  |
| 1. Adams MM 1997 [27] | 11.7% of mothers with less than 12 years of education were missing key linkage variables v. 3.2% for those with 13-15 years. |
| 2. Blakely T 2000 [37] | Those in the most deprived SES decile had a risk ratio for linkage of 0.92 (95%CI: 0.90-0.94) compared to the least deprived decile. This effect was constant across different causes of death. |
| 3. Harris T 2005 [21] | 10.3% of people with no car available did not consent to having their records linked v. 6% of those with a car (Odds Ratio for consent=1.8, 95% CI: 1.2-2.6). |
| 4. Holian J 2000 [36] | 42.4% were low SES children among linked records v 75% unlinked due to higher rates of unregistered births. |
| 5. Huang N 2007 [25] | Illiterate (21.1%) and lowest income groups (14.9%) did not consent to record linkage v. college educated (9.9%) and highest income groups (9.9%). |
| 6. Klassen AF 2005 [47] | Parents within the $30-49,999 p.a. income group had odds of consenting to linkage of 0.3 (95% CI: 0.2-0.7) v. those earning $80K plus. |
| 7. Maizlish NA 2005 [34] | The sensitivity of matching was highest among people with commercial medical insurance at 94.4% v. 91.7% for people with Medicaid (p<0.05). |
| 8. Nitsch D 2006 [41] | 69% of children in the linked cohort had fathers with manual occupations compared to 64% in the unlinked cohort. This difference was not found to be significant. |
| 9. Tate AR 2006 [26] | 12.5% of those who never worked or were long-term unemployed did not consent to linkage v. 5.4% of those in managerial or professional positions. |
| 10. Waller DK 1996 [23] | Proportions were not reported but distributions of Medicaid recipients versus private insurance were noted to be identical in matched compared to unmatched records. |
| 11. Young AF 2001 [48] | Those who consented to record linkage in all age groups tended to have higher levels of education (Post-school education v. Not: Young Women 69% v 59%, Middle-aged women 37% v 27% and Older women 18% v 12%). |
| Health Status |  |
| 1. Adams MM 1997 [27] | 10.5% of babies born with low birth weights (<1500 g) had mothers missing key linkage variables compared to 5.9% for those weighing 2500 g and over. |
| 2. Baker R 2000 [19] | Mean severity score was 79.1 in patients with asthma that consented to linkage compared to 77.0 for those that did not. Mean severity score was 85.0 in patients with angina that consented to linkage compared to 85.1 for those that did not. This was noted not to be a significant difference. |
| 3. Cryer 2001 [31] | People with serious injuries linkage rates of 66% v. 61% overall due to treatment in hospital and a higher rate of reporting accidents to the police. |
| 4. Dalrymple AJ 1994 [42] | Misidentification by unique ID was 22% among patients in the community sector v <0.5% treated in psychiatric hospitals in the first linkage run. |
| 5. Dunn KM, 2004 [24] | People with the symptom under investigation had a pooled odds ratio for consent to medical record linkage of 1.44 (95% CI: 1.34, 1.55) v. controls. |
| 6. Ford JB 2006 [20] | 26.3% were pre-term births among unlinked records v 6.5% in the linked cohort. 79.5% were induced pregnancies in unlinked records v 63.2% linked. |
| 7. Grace KR 1995 [52] | 5.2% of unlinked records had major birth defects v 1% in the linked cohort. 31% in unlinked records were premature v 4.7% in linked cohort due to incomplete identifying information. |
| 8. Harris T 2005 [21] | People with long-standing illnesses had non-consent rates of 5.2% v. 10.2% for those without. People with 3 or more diagnosed illnesses had non-consent rates of 3.6% v. 11.6% for those with none. |
| 9. Herrchen B 1997 [50] | There were 0.85% very low birth weight newborns in the linked data compared to 0.84% overall and 4.08% moderately low birth weight newborns in the linked data compared to 4.09% overall. These differences were not thought to be significant. |
| 10. Holian J 2000 [36] | Approximately 10% of pre-term and low birth weight babies who died were not registered and therefore not linked compared to 0% of post-neonate deaths. |
| 11. Huang N 2007 [25] | The mean general health score on the SF-36 was 72.6 for people who consented compared to 70.3 for those that did not. The adjusted odds ratio for the general health score was 1.02 (95% CI: (0.98 – 1.06) for those that did not consent to linkage. |
| 12. Kariminia A 2005 [54] | The sensitivity of the linkage was 92.3% for drug-related deaths and 93.8% for suicides, all other causes of death were >98%. |
| 13. Klassen AF 2005 [47] | Parents of sicker children had higher odds of consenting to linkage (OR=1.5, 95% CI:1.2-2.0) versus those without a health problem. |
| 14. Liu S 1999 [53] | 38.6% of unlinked records included babies with jaundice v. 40.9% of linked records (p<0.05).  6.4% of unlinked records included babies with a low birth weight (<2500 g) v. 5.6% of linked records (p<0.01) due to missing or incomplete information. |
| 15. Magliano D 2003 [55] | The sensitivity of matching for deaths related to cancer (95.2%) was higher than deaths related to cardiovascular disease (92.5%) |
| 16. Rosman DL 1994 [40] | More severe injuries (AIS of 5) had linkage rate of 78.9% v. 40% for non-trauma cases due to treatment in hospital, which had more complete reporting rates due legal obligations to report the accident. |
| 17. Sunderam S 2006 [45] | Babies with low birth weights (<1500 g) had linkage rates of 84.2% v. 92% for those with a moderately low weight (1500-2500 g) and 90% for those with a normal weight (>2500g) (p<0.01) due to lower reporting rates of very early neonate deaths. |
| 18. Tromp M 2006 [51] | The accuracy of singleton babies with readmissions was the same as that of those with only one admission. However, twin births with readmissions were noted to have higher error rates. |

*Studies are reported under each relevant heading, so some studies appear more than once.
