# Supplementary material for: Data Linkage: A powerful research tool with potential problems
Source: BMC Health Serv Res. 2010 Dec 22;10:346. doi: 10.1186/1472-6963-10-346 (PMC3271236; doi:10.1186/1472-6963-10-346)
Supplement: Additional file 4 — A framework for evaluating data linkage studies. Reasons for unlinked records and suggested reporting items for studies utilising data linkage to identify potential quality issues. [file 1472-6963-10-346-S4.DOC]

Additional File 4. A framework for evaluating data linkage studies

| Completeness of source databases |
| --- |
| *This may include under-ascertainment of cases or differences in reporting practices and/or inclusion criteria between the data sources to be linked* |
| 1. A description of the data sources to be used in the study should be included. |
| 2. The number of eligible records obtained from each data-set and the reasons for differences, if any, should be reported. |
| Accuracy of data sources |
| *Incomplete/inaccurate data, especially if there is variation between sites or groups of patients* |
| 1. Variables selected by researchers for linkage and analysis should be reported. |
| 2. The completion rate and accuracy of variables to be linked should be presented. |
| 3. Coding practices and the use of standardised definitions should be stated, if used. |
| Linkage methodology and technology |
| *Linkage algorithm (i.e. deterministic or probabilistic), technology and linkage analysts used may affect the overall linkage rate.* |
| 1. A measure of the validity of the linked data-sets (e.g. false positive and false negative rates, if available) should be given. |
| 2. An analysis of potential sources of bias among non-linked cases should be reported. |
| 3. The denominator used to derive linkage rates and justification for this should be reported. |
| 4. A description of the data linkage methods (i.e. deterministic or probabilistic) with a justification for these. |
| 5. Methods used to reduce the number of non-linking records should be described, if used (Optional) |
| Ethical and data security considerations |
| 1. Opt-in inclusion involving one or more of the data sources should be presented, if it was required. |
| 2. Linkage consent practices (if consent-based) should be described. |
